# Polyphenols as Lung Cancer Chemopreventive Agents by Targeting microRNAs

**DOI:** 10.3390/molecules27185903

**Published:** 2022-09-11

**Authors:** Jing Li, Xianmei Zhong, Yueshui Zhao, Jing Shen, Chalermchai Pilapong, Zhangang Xiao

**Affiliations:** 1 Department of Oncology and Hematology, The Affiliated Traditional Chinese Medicine Hospital of Southwest Medical University, Luzhou 646000, China; 2 Center of Excellence for Molecular Imaging (CEMI), Department of Radiologic Technology, Faculty of Associated Medical Sciences, Chiang Mai University, Chiang Mai 50200, Thailand; 3 Laboratory of Molecular Pharmacology, Department of Pharmacology, School of Pharmacy, Southwest Medical University, Luzhou 646000, China; 4 Cell Therapy & Cell Drugs of Luzhou Key Laboratory, Southwest Medical University, Luzhou 646000, China; 5 South Sichuan Institute of Translational Medicine, Luzhou 646000, China; 6 Department of Pharmacy, People’s Hospital of Nanbu County, Nanchong 637300, China; 7 Center of Radiation Research and Medical Imaging, Department of Radiologic Technology, Faculty of Associated Medical Sciences, Chiang Mai University, Chiang Mai 50200, Thailand

**Keywords:** polyphenol, lung cancer, microRNA

## Abstract

Lung cancer is the second leading cause of cancer-related death worldwide. In recent decades, investigators have found that microRNAs, a group of non-coding RNAs, are abnormally expressed in lung cancer, and play important roles in the initiation and progression of lung cancer. These microRNAs have been used as biomarkers and potential therapeutic targets of lung cancer. Polyphenols are natural and bioactive chemicals that are synthesized by plants, and have promising anticancer effects against several kinds of cancer, including lung cancer. Recent studies identified that polyphenols exert their anticancer effects by regulating the expression levels of microRNAs in lung cancer. Targeting microRNAs using polyphenols may provide a novel strategy for the prevention and treatment of lung cancer. In this review, we reviewed the effects of polyphenols on oncogenic and tumor-suppressive microRNAs in lung cancer. We also reviewed and discussed the potential clinical application of polyphenol-regulated microRNAs in lung cancer treatment.

## 1. Introduction

Lung cancer originates from the bronchial mucosa or glands of the lung. Lung cancer can be mainly divided into non-small cell lung cancer (NSCLC) and small cell lung cancer (SCLC). Among all lung cancers, non-small cell lung cancer accounts for about 85–88%, while small cell lung cancer accounts for about 12–15% [1]. According to the reports of the World Health Organization, the incidence rate of lung cancer in 2020 was 22.4 cases per 100,000 people, ranking second in terms of cancers; while the mortality rate of lung cancer is as high as 18 cases per 100,000 people (https://www.wcrf.org/cancer-trends/lung-cancer-statistics/ (accessed on 23 March 2022)). The existing treatment methods of lung cancer are mainly surgery, chemotherapy, and radiotherapy. These treatment methods have serious side effects and easily cause discomfort. Herb and plant derived-chemicals have the characteristics of less toxicity and side effects, showing better therapeutic effects, and can improve the quality of life of patients and weaken the deficiencies of existing therapeutic drugs [2].

Polyphenols are widely found in foods and beverages of plant origin (e.g., fruits, vegetables, spices, soybeans, nuts, tea, wine, etc.) [3,4]. Polyphenols are a group of plant components, with multiple hydroxyl phenols existing in plants. They are important secondary metabolites in plants and have a polyphenol structure. Polyphenols are mainly synthesized from shikimic acid and malonic acid [5]. Polyphenols have physiological functions, having roles in antioxidation, the prevention of cardiovascular disease, anticancer activities, and the inhibition of microorganisms [6,7,8,9,10,11,12,13,14]. Polyphenols show cancer-preventive effects by regulating diverse signaling pathways or biological processes, including inducting apoptosis, stimulating immune cell functions, and causing anti-inflammatory effects [15].

Over the past decade, the role of non-coding RNAs (ncRNAs) in carcinogenesis and the use of ncRNAs as targets for tumor inhibition have been hot study topics. NcRNAs are divided into three categories in terms of length: small non-coding RNAs of less than 50 nucleotides (nt), including microRNAs, siRNAs, and piRNAs; medium non-coding RNAs of 50 nt to 500 nt, including rRNA, tRNA, snRNA, snoRNA, SLRNA, and SRPRNA; long non-coding RNAs greater than 500 nt, including long non-coding RNAs without a PolyA tail. NcRNAs can be transcribed from the genome, but they can perform their biological functions at the RNA level without being translated into proteins [16]. At present, studies have found that ncRNAs are involved in the occurrence and development of a variety of cancers. microRNA, a 22-nucleotide RNA molecule, is an endogenous single-stranded RNA that reduces gene expression through the RNA interference; that is, its own target gene expression product [17]. More and more data are showing that the miRNAs are involved in the progression of lung cancer [18]. This provides a new way to find more effective drugs for the treatment of lung cancer.

Recent studies have shown that miRNAs are involved in polyphenol-induced carcinogenesis inhibition. Here, we reviewed the functions of polyphenols in oncogenic and tumor-suppressive microRNAs in lung cancer. We also reviewed and discussed the potential application of polyphenol-regulated microRNAs in lung cancer treatment.

## 2. Classification of Polyphenols

Polyphenols are a group of phenolic chemicals with a basic phenolic ring [19]. According to the strength of the phenolic ring, the polyphenols can be roughly divided into five categories (Table 1) [20]. The first category is phenolic acids, including gallic acid [21]. The second category is stilbenes, including resveratrol [22]. The third category is flavonoids, including EGCG, quercetin, genistein, kaempferol, and baicalin [23]. The fourth category is lignans, including honokiol [24]. The fifth category is curcuminoids, including curcumin [25].

## 3. Lung Cancer and microRNA

The occurrence of lung cancer is mainly the result of the interactions of environmental factors and genetic factors. Recent studies have shown that in addition to the abnormal expression of some signal pathways and oncogenes, lung cancer is also associated with an imbalance in microRNA expression [26].

**Figure 1:**
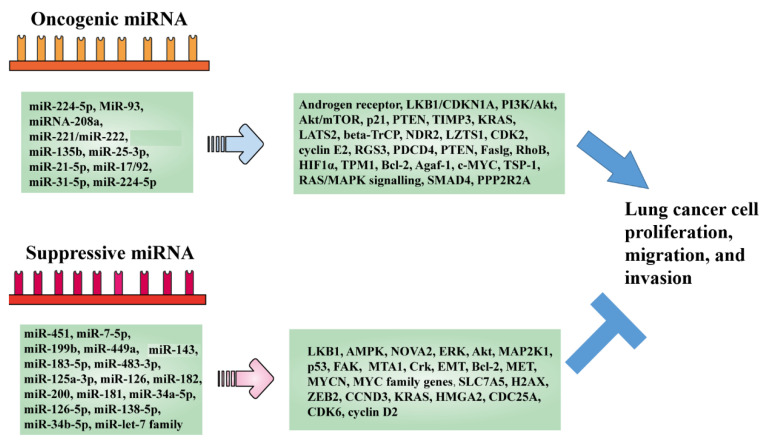
Oncogenic and tumor-suppressive microRNAs in lung cancer. miRNAs act as cancer promoters/inhibitors by targeting/regulating relevant proteins/pathways in lung cancer.

The expression of miRNAs is complex. Some miRNAs are upregulated in tumors and play a role similar to oncogenes, while some miRNAs are downregulated in tumors and play the role of tumor suppressor genes. An abnormal miRNA molecule can affect the expression of hundreds of miRNAs. When an miRNA regulates key genes, it will have a great impact on the cell function [27]. In the pathogenesis and progression of SCLC and NSCLC, some miRNAs have been speculated as oncogenes, tumor suppressor genes, and cancer progression (Table 2 and Figure 1).

In recent years, studies have shown that miR-224-5p induces the migration, invasion, and proliferation of NSCLC [28]. Furthermore, miR-93 promotes NSCLC metastasis by inhibiting LKB1/CDKN1A to activate the PI3K/Akt pathway [29]. Another miRNA with tumor-promoting activity is miR-208a, which promotes the activation of Akt/mTOR in NSCLC cells through p21, and then promotes the proliferation of tumor cells [30]. Additionally, miR-221/miR-222 promotes cell migration through the target genes PTEN and TIMP3 [31]. A recent study reported that miR-135b was upregulated in highly invasive NSCLC, while miR-135b inhibited the growth and invasion of mouse lung tumors [32]. In SCLC and NSCLC, many miRNAs are speculated to have carcinogenic effects, such as miR-25-3p, miR-21-5p, miR-17/92, miR-31-5p, and miR-224-5p [34,35,36,37,38,39,40,41].

On the other hand, many miRNAs have a tumor-suppressive function in lung cancer. For example, miR-451 can inhibit the proliferation and migration of NSCLC cells by regulating LKB1/AMPK [42,43]. Moreover, miR-143 inhibits cell growth by inhibiting k-RAS translation [33], while miR-7-5p inhibits the tumor metastasis of non-small cell lung cancer by targeting NOVA2 [44]. A recent study showed that miRNA-199b targeted ERK and Akt signaling pathways and inhibited the proliferation and metastasis of NSCLC [45]. Moreover, miRNA-449a inhibits the invasion of NSCLC cells by inhibiting MAP2K1 [46], while miR-183-5p inhibits p53, thereby promoting the metastasis of NSCLC [47]. Additionally, miR-483-3p can target integrin β3 to inhibit the FAK/ERK signaling pathway and the invasion and migration of drug-resistant lung cancer cells [48]. Furthermore, miR-125a-3p inhibits the proliferation and infiltration of non-small cell lung cancer cells by downregulating MTA1 [49]. When the expression of miR-126 and mir-182 was upregulated, the expression of Crk protein decreased, and the migration, infiltration and adhesion of tumor cells were inhibited [50,51]. The high expression of miR-200 can inhibit the ability of epithelial mesenchymal transformation, invasion and metastasis of metastatic lung adenocarcinoma cells [52]. The experiment of NSCLC cell line A549 showed that upregulating miR-181 could significantly inhibit cell growth, migration and induce apoptosis [53]. In addition, miR-181 inhibition was found to be associated with higher Bcl-2 levels [32]. miR-34a-5p, miR-126-5p, miR-138-5p, miR-34b-5p, miR-let-7 family, etc. in SCLC and NSCLC were speculated to be miRNA suppressor [7,8,9,10,11,12,13,14,37].

## 4. The Role of Polyphenols in Lung Cancer by Targeting microRNAs

**Figure 2:**
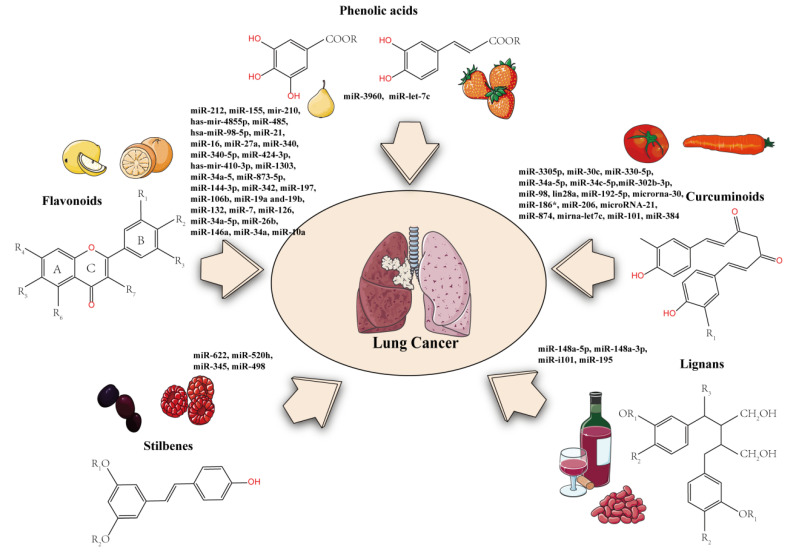
Modulation of microRNA expression using polyphenols. Polyphenols inhibit the growth of lung cancer by up- or downregulating related microRNAs.

More and more data show that miRNA is involved in the progression of lung cancer. It provides a new way to find more effective drugs for the treatment of lung cancer. Recent studies have shown that polyphenols play a pharmacological role in lung cancer by regulating miRNAs (Table 3 and Figure 2).

### 4.1. Flavonoids

Epigalocatechin gallate (EGCG), the main component of green tea polyphenols, is a catechin monomer isolated from tea [54]. Studies showed that the expression levels of miR-212 were decreased and the expression of miR-155 were increased in EGCG-treated A549 by regulating the MAPK signaling pathway, which in turn inhibited cancer cell proliferation and migration [55]. Wang et al. found that EGCG, through the upregulation of HIF-1α and the expression of mir-210, inhibited the growth of lung cancer cells [56]. At the same time, EGCG can enhance the expression of has-miR-4855p, significantly inhibit the growth of NSCLC cells, and induce apoptosis [57]. Another study showed that EGCG inhibited cancer stem cell-like properties by upregulating the expression of miR-485 and reducing the expression of CD44 [58]. Meanwhile, some studies have found that EGCG can inhibit the expression of hsa-miR-98-5p and upregulate the expression of p53, thereby enhancing the efficacy effects of cisplatin on A549 cells [59]. Skullcapflavone I is a natural product found in Scutellaria baicalensis, Andrographis paniculata, and other organisms [60]. Skullcapflavone I can downregulate the expression levels of miR-21, enhance the expression levels of PP2A in A549 cells, and inhibit the proliferation of human lung cancer cells [61]. Quercetin is a widely distributed flavonoid alcohol compound with a variety of biological activities in plants [62]. Studies found that the expression level of miR-16 was upregulated with quercetin treatment, in turn mediating the decrease in Claudin-2 expression and inhibiting the invasion and migration of lung adenocarcinoma cells [55,63]. Genistein is a soybean isoflavone and phytoestrogen with antitumor activity [64]. Genistein-treated A459 cells showed decreased expression of miR-27a and increased expression of MET, which in turn promoted the apoptosis of A459 cells [55].

Kaempferol is an organic compound with the chemical formula c15h10o6 and is a flavonoid. After kaempferol treatment, the expression of mir-340 increased, the expression of the target gene cyclin D1 decreased, and the expression of PTEN increased, which inhibited proliferation and promoted the apoptosis of A549 cells [55]. Similarly, Han et al. found that the expression of mir-340 was upregulated, the level of PTEN increased, the phosphorylated levels of PI3K and AKT were decreased, the proliferation of A549 cells was inhibited, and the apoptosis and autophagy of A549 cells were increased after kaempferol treatment compared with the control group [65]. Baicalin is a flavonoid extracted and isolated from the dried roots of Scutellaria baicalensis Georgi, a dicotyledonous Labiatae plant [66]. Recent studies found that the expression levels of miR-340-5p and the target gene NET1 were increased after baicalin treatment, in turn inhibiting the proliferation and invasiveness of A549 and H1299 cells [67]. Meanwhile, Baicalein inhibited cell growth and increased the sensitivity of A549 and H460 cells to cisplatin through the miR-424-3p-targeted PTEN/PI3K/AKT pathway [68]. The Radix Tetrastigma hemsleyani flavone (RTHF) is extracted from a traditional Chinese medicinal herb *T. hemsleyani* [69]. The increase in has-miR-410-3p in A549 cells caused by RTHF may play a role in the inhibition of A549 cells via downregulating the expression of MMP14 and MMP16 [69]. Moreover, the downregulation of miR-1303 by RTHF may occur through targeting CLDN18, GSK3β, and SFRP1, thereby inhibiting the proliferation, migration, and invasion of A549 cells [70]. Apigenin mainly exists in Daphneceae, Verbenaceae, and Selaginellaceae plants, especially in celery [71]. It was found that apigenin may induce apoptosis by upregulating miR-34a-5p in A549 cells and downregulating SNAI1 [72].

The soy isoflavone genistein is usually present in genistein and daidzein. It is a bioflavonoid in soybean products and other plants [73]. In NSCLC cells treated with the soy isoflavone genistein, miR-873-5p inhibited cell proliferation, migration, and invasion and increased apoptosis by regulating FOXM1 [74]. Licochalcone A (Lico A) is a post chalcone isolated from the root of Glycyrrhiza uralensis, a plant from Xinjiang Province in China [75]. It is reported that LiCo A can significantly promote the expression of miR-144-3p, downregulate the expression levels of Nrf 2, and finally induce apoptosis in lung cancer cells [76]. Chen et al. also found that Lico A can activate the unfolded protein response (UPR) and induce autophagy in H292 cells, thereby inducing apoptosis [77]. Puerarin is a C-glycosyl compound and a hydroxyisoflavone [78]. Purerin inhibits the expression of CCND1 by upregulating miR-342; inhibits cell viability, migration, invasion, and the cell cycle process; and enhances the apoptosis of NSCLC cells [79]. Nobiletin is a natural product found in Ageratum conyzoides and Viburnum tinus [80]. Sp et al. found that nobiletin inhibited the expression of PD-L1 through the EGFR/JAK2/STAT3 signaling pathway, while the expression levels of STAT3 and PD-L1 were regulated by miR-197, thereby enhancing the antitumor immunity [81]. Recent studies have shown that the downregulation of miR-106b by grape seed procyanidin (GSE) induced the expression levels of tumor inhibition cycle-independent kinase inhibitor 1A (CDKN1A) and p21, which further promotes the antitumor effect of GSE [82]. Another study found that grape seed procyanidin significantly downregulated the expression of miR-19a and-19b in tumor cells, increased the mRNA and protein levels of insulin-like growth factor II receptor (IGF-2R) and phosphatase and tensin homologue (PTEN), and significantly inhibited tumor growth [83].

Hesperidin is a flavanone glycoside, which is found in citrus fruits [84]. Hesperidin can promote the apoptosis of lung cancer cells by increasing the expression of miR-132 and reducing the expression of ZEB2, so as to inhibit the proliferation of lung cancer cells [85]. Breviscapine is found in Indian wood, perilla, and other organisms [86]. Zeng et al. found that breviscapine enhanced the expression of miR-7, upregulated Bax/Bcl-2, and promoted apoptosis [87]. It was found that Nepeta cataria L. extract can regulate the expression of miR-126 and regulate the PI3K-Akt signaling pathway to perform the anticancer effect [88]. Luteolin is a natural product found in Cryptomeria japonica and Epimedium [89]. Luteolin upregulates the expression of miR-34a-5p by targeting MDM4, inhibits tumorigenesis, and induces the apoptosis of NSCLC cells [90]. Orientin is a C-glycosyl compound, and it is believed that orientin regulates the expression of COX-2/PGE-2 in the A549 cell line through miR-26b and miR-146a and reduces the proliferation, migration, and invasion of A549 cells [91]. Rhamnetin is a natural product found in Liriodendron tulipifera and Albizia julibrissin [92]. Cirsiliol is a natural product found in Salvia lineata and Teucrium chamaedrys. Rhamnetin and Cirsiliol can inhibit the EMT of lung cancer cells through the miR-34a-mediated downregulation of Notch-1 expression [93]. Icaritin exists in Epimedium bicolor, Epimedium aculeatum, and Epimedium wushanese [94]. Icaritin inhibits NSCLC cell proliferation by downregulating miR-10a, which could regulate the expression of PTEN [95].

### 4.2. Phenolic Acids

Caffeic acid phenethyl ester (CAPE) is a natural product found in Euonymus alatus and Alibertia macrophylla, and is the phenethyl alcohol ester of caffeic acid and a bioactive component of honeybee hive propolis, with antineoplastic, cytoprotective, and immunomodulating activities [96]. Mo et al. found that CAPE treatment downregulated the expression of YAP1 and C-MYC, thereby inducing H446 cell apoptosis. Moreover, they found that miR-3960 upregulated the expression of C-MYC and participated in CAPE-induced SCLC cell apoptosis [97]. Cucurbitacin B is a cucurbitacin derived from the hydrides of lanosterol [98]. Cucurbitacin B inhibits the proliferation and promotes the apoptosis of lung cancer cells through the lncRNA XIST/miR-let-7c axis [99].

### 4.3. Stilbenes

Resveratrol, a non-flavonoid polyphenol organic compound, is an antitoxin produced by many plants when stimulated [100]. In lung cancer cells treated with resveratrol, cell proliferation was inhibited via the miR-622/k-Ras axis [101]. Moreover, resveratrol can also inhibit the expression of FOXC2 and tumor activity by regulating the miR-520h-mediated signal cascade [102]. Lu et al. found that resveratrol inhibited NSCLC cell proliferation via miR-345- and miR-498-regulated MAPK/CFO and Akt/BCL2 signaling pathways by directly targeting MAPK1 and PIK3R1, respectively, which increased the sensitivity of NSCLC cells to gefitinib and induced apoptosis [103].

### 4.4. Lignans

Honokiol is found in Cryptomeria fortunei, star anise, and other organisms [104]. Honokiol inhibited the proliferation and migration of NSCLC cells and induced the apoptosis of NSCLC cells through miR-148a-5p and miR-148a-3p, probably by targeting ERBB3 and itga5 through the PI3K/Akt signaling pathway [105]. Treatment with Phyllanthus emblica L (PEL) extract could effectively prevent precancerous lesions of lung cancer by regulating the IL-1β/miR-101/LIN28B signaling pathway [106]. Ailanthone comes from Ailanthus altissima, and can inhibit the proliferation of lung cancer cells and promote the apoptosis and autophagy of lung cancer cells [107]. Hou et al. found that Ailanthone induced the apoptosis and autophagy of lung cancer cells by upregulating the expression of miR-195 [108].

### 4.5. Curcuminoids

Curcumin is a natural nutrient compound derived from long Jiang Huang (Jiang Huang), and shows good pharmacological effects, including anti-inflammatory, neuroprotective, and antidiabetic effects [109]. Curcumin-induced miR-3305p upregulation in lung cancer cells was inversely related to the metastasis of lung cancer cells and reduced their invasion; meanwhile, curcumin upregulates miR-30c expression, which in turn reduces the expression of MTA1 to improve the sensitivity of NSCLC cells to PTX chemotherapy [110]. Similarly, Zhan et al. found that the expression of miR-330-5p was significantly upregulated in lung cancer cells, and the antimigration effect of curcumin was mediated by miR-330-5p [97]. Another study found that curcumin may inhibit lung cancer metastasis by miR-34a-5p/miR-34c-5p/miR-302b-3p-lef1-ccnd1/Wnt1/MYC axis [111]. Liu et al. studied the effect of curcumin on the expression of miR-98 in lung cancer cells. They found that the expression of miR-98 was upregulated by curcumin treatment and inhibited the migration and invasion of lung cancer cells by inhibiting the expression of MMP2 and MMP9 induced by lin28a [112]. ATP1B1 (β1 subunit of Na^+^/K^+^-ATPase) is a target of miR-192-5p [113]. It was found that curcumin promotes the apoptosis of NSCLC cells through the p53-miR-192-5p/215-XIAP and PI3K/Akt signaling pathways [114,115]. Moreover, curcumin promoted an increase in miR-192-5p expression level in a dose-dependent manner and with a decrease in c-MYC expression [113,116]. Finally, curcumin can inhibit the proliferation, migration, invasion, and viability of NSCLC cells in a dose-dependent manner [116]. Curcumin increases the sensitivity of paclitaxel-resistant NSCLC cells to paclitaxel through a reduction in MTA1 mediated by miR-30 [117]. Curcumin can also significantly downregulate the expression of miR-186* in A549/DDP cells and promote the apoptosis of A549/DDP cells [118,119]. Wang et al. found that curcumin inhibited the migration and invasion of NSCLC cells by upregulating miR-206 expression and by inhibiting the PI3K/Akt/mTOR signaling pathway [120]. It was also found that the protein level of PTEN, the putative target of miR-21, was significantly increased in curcumin-treated A549 cells, showing antiproliferation and proapoptotic activities in NSCLC cells [121]. It was found that curcumin inhibited the expression and activity of MMP-2 by upregulating miR-874 in A549 cells. Curcumin can also upregulate miR-let7c and miR-101 in A549 cells [122]. Zeste homolog 2 was significantly downregulated when A549 cells overexpressed miRNA-let7c and miR-101 [122]. The study speculated that the effect of curcumin on the miRNA may lead to the inhibition of the growth of lung cancer cells [122]. Curcumin inhibits the growth of NSCLC by downregulating CIRC PRKCA, while PRKCA regulates the expression of ITGB1 via miR-384 [123].

## 5. Clinical Trials Using Polyphenols for Lung Cancer Treatment

To date, there have been 12 clinical trials of polyphenols in lung cancer (http://clinicaltrials.gov/ (accessed on 8 August 2022), listed in Table 4). Among these clinical trials, flavonoids are the major ones used for treatment. Zhao et al. studied the side effects and optimal dose of EGCG in patients with non-small cell lung cancer. The initial dose of EGCG was 400 mg administered twice a day. The second incremental dose was 800 mg, the third incremental dose was 1200 mg, the fourth incremental dose was 1600 mg, and the fifth incremental dose was 2000 mg (NCT01317953). The results showed that oral EGCG is feasible, safe, and effective, and the recommended concentration of EGCG in patients with non-small cell lung cancer in the second stage of treatment is 440 μM [124]. Scott et al. determined that the maximum tolerated dose of green tea extract in patients with advanced lung cancer was 3 g/m^2^/day. At this dose, the green tea extract was well tolerated and the toxicity was no more than grade 3 or 4 [125]. Siegenthaler et al. found that flavor aesthetic acid (NSC.347512, LM975) had slight antitumor activity against NSCLC [126]. However, the results of most clinical trials have not been published. Therefore, whether polyphenols mediate antitumor effects through miRNAs in clinical trials has not been clarified.

## 6. Conclusions and Future Perspectives

In the past two decades, miRNAs have been proven to play a major role in the pathogenesis of lung cancer and have become candidate therapeutic targets. Preclinical studies have shown that polyphenols can downregulate pro-tumor-associated microRNAs or upregulate tumor-associated microRNAs, thereby exerting their antitumor function in lung cancer. However, the therapeutic effects of using miRNAs for lung cancer treatment need to be demonstrated in clinical trials. Thus, further studies are needed to explore this promising field. Therefore, in future clinical trials, we could study the effects of polyphenols on miRNAs in lung cancer patients in vivo by using new technologies such as metabolomics and single-cell sequencing. Special attention should be paid to the cancer-promoting or cancer-suppressing miRNAs that were found to be affected by polyphenols in preclinical experiments. We could screen for different polyphenols targeting specific types of miRNAs associated with cancer through the application of polyphenols in clinical settings.

## Figures and Tables

**Table 1:** Classification of polyphenols.

Classification	Chemicals
Phenolic Acids	Caffeic Acid Phenethyl Ester, Cucurbitacin B
Stilbenes	Resveratrol
Flavonoids	EGCG, Skullcapflflavone I, Quercetin, Genistein, Kaempferol, Baicalin, Radix Tetrastigma hemsleyani flavone, Apigenin, Soy isoflavone genistein, Licochalcone A, Puerarin, Nobiletin, Grape seed procyanidin, Hesperidin, Breviscapine, Nepeta cataria L.’s extract, Luteolin, Orientin, Rhamnetin, Cirsiliol, Icaritin
Lignans	Honokiol, Phyllanthus emblica L, Ailanthone
Curcuminoids	Curcumin

**Table 2:** Oncogenic and tumor-suppressive miRNAs in lung cancer.

	miRNA Name	Targets/Regulators	Reference
Oncogenic miRNAs	miR-224-5p	Androgen receptor	[28]
	miR-93	LKB1/CDKN1A, PI3K/Akt	[29]
	miRNA-208a	Akt/mTOR, p21	[30]
	miR-221/miR-222	PTEN, TIMP3	[31]
	miR-135b	LATS2, beta-TrCP, NDR2 and LZTS1	[32,33]
	miR-25-3p	CDK2, cyclin E2, RGS3	[32,34]
	miR-21-5p	PDCD4, PTEN, Faslg, RhoB, HIF1α, TPM1, Bcl-2L	[35,36,37]
	miR-17/92	Agaf-1, c-MYC, PTEN, p21	[37,38,39]
	miR-31-5p	TSP-1, RAS/MAPK signalling	[37,40]
	miR-224-5p	LATS2, SMAD4, PPP2R2A	[37,41]
	miR-451	LKB1/AMPK	[37,42,43]
Tumor Suppressive miRNAs	miR-143	KRAS	[33,42,43]
	miR-7-5p	NOVA2	[44]
	miR-199b	ERK, Akt	[44,45]
	miR-449a	MAP2K1	[45,46]
	miR-183-5p	p53	[46,47]
	miR-483-3p	FAK/ERK	[47,48]
	miR-125a-3p	MTA1	[48,49]
	miR-126, miR-182	Crk	[49,50,51]
	miR-200	EMT	[50,51,52]
	miR-181	Bcl-2	[32,52]
	miR-34a-5p	BCL-2, MYC, MET, MYCN, p53	[7,8,32]
	miR-126-5p	SLC7A5	[9,37]
	miR-138-5p	H2AX, ZEB2, CCND3	[10,11,12,37]
	miR-34b-5p	BCL-2, MYC, MET	[13,37]
	miR-let-7 family	KRAS, MYC, HMGA2, CDC25A, CDK6, cyclin D2	[14,37]

**Notes:** miR means mature miRNA; to distinguish miRNAs, numbers and letters were used; 5p and 3p mean the mature miRNA comes from the 5′ and 3′ arms of the precursor miRNA, respectively.

**Table 3:** Modulation of microRNA expression by polyphenols.

Natural Compound	ncRNA	Targets/Regulators	Cell Processes	Reference
EGCG	miR-212 (↓), miR-155 (↑), mir-210 (↑), has-miR-4855p (↑), miR-485 (↑), hsa-miR-98-5p (↓)	MAPK signaling pathway, HIF-1α, CD44, p53	Inhibition of cell proliferation, migration, apoptosis and growth of lung cancer cells, enhancing the efficacy of cisplatin in A549 cells	[55,56,57,58,59]
Skullcapflflavone I	miR-21 (↓)	PP2A	Inhibition of cell proliferation	[61]
Quercetin	miR-16 (↑)	Claudin-2	Destroyed the invasion and migration of lung adenocarcinoma cells	[55,63]
Genistein	miR-27a (↓)	MET	Promoted apoptosis	[55]
Kaempferol	miR-340 (↑)	Cyclin D1, PTEN, PI3K, AKT	Cell apoptosis, inhibition of proliferation, autophagy increased	[55,65]
Baicalin	miR-340-5p (↑), miR-424-3p (↓)	NET1, PTEN/PI3K/Akt pathway	Inhibition of proliferation and invasiveness	[67,68]
Radix Tetrastigma Hemsleyani Flavone	has-mir-410-3p (↑)	RTHF, MMP14, MMP16, CLDN18, GSK3β, SFRP1	Inhibition of proliferation, migration, and invasion	[70]
Apigenin	miR-34a-5 (↑)	SNAI1	Induced apoptosis	[72]
Soy Isoflavone Genistein	miR-873-5p (↑)	FOXM1	Inhibited cell proliferation, migration, and invasion and increased apoptosis	[74]
Licochalcone A	miR-144-3p (↑)	NRF2, unfolded protein response	Induced apoptosis and autophagy	[76,77]
Puerarin	miR-342 (↑)	CCND1	Inhibition of cell viability, migration, invasion, and cell cycle process, enhancement of the apoptosis	[79]
Nobiletin	miR-197 (↓)	PD-L1, EGFR/JAK2/STAT3 signaling pathway,	Enhanced antitumor immunity	[81]
Grape seed procyanidin	miR-106b (↓), miR-19a (↓), miR-19b (↓)	CDKN1A, insulin-like growth factor II receptor, PTEN	Inhibition of tumor growth	[83]
Hesperidin	miR-132 (↑)	ZEB2	Inhibition of the proliferation	[85]
Breviscapine	miR-7 (↑)	Bax/Bcl-2	Promoted apoptosis	[87]
Nepeta Cataria L.’s Extract	miR-126 (↑)	PI3K-Akt signaling pathway	Anticancer effect	[88]
Luteolin	miR-34a-5p (↑)	MDM4	Inhibition of tumorigenesis and induces apoptosis	[90]
Orientin	miR-26b (↑), miR-146a (↑)	COX-2/PGE-2	Reduces cell proliferation, migration and invasion	[91]
Rhamnetin, Cirsiliol	miR-34a (↑)	Notch-1	Inhibition of EMT	[93]
Icaritin	miR-10a (↓)	PTEN	Antitumor effect	[95]
Caffeic Acid Phenethyl Ester	miR-3960 (↑)	YAP1, C-MYC	Cell apoptosis	[97]
Cucurbitacin B	LncRNAXIST (↓), miR-let-7c (↑)	IL-6/STAT3 pathway	Inhibition of the proliferation and promote apoptosis	[99]
Resveratrol	miR-622 (↑), miRNA-520h (↓), miR-345 (↑), miR-498 (↑), ak001796 (↓)	K-RAS, FOXC2, MAPK/CFOs, Akt/BCL2 signaling pathways	Induced apoptosis	[101,102,103]
Honokiol	miR-148a-5p (↑), miR-148a-3p (↑)	ERBB3 and ITGA5, PI3K/Akt signaling pathway	Inhibited proliferation and migration	[105]
Phyllanthus Emblica L	miR-101 (↑)	IL-1β/MiR-101/LIN28B signaling pathway	Effectively prevented precancerous lesions	[106]
Ailanthone	miR-195 (↑)	PI3K, Akt, Jak, STAT3	Induced apoptosis and autophagy	[108]
Curcumin	miR-3305p (↑), miR-30c (↑), miR-330-5p (↑), miR-34a-5p (↑), miR-34c-5p (↑), miR-302b-3p (↑), miR-98 (↑), miR-192-5p (↑), miR-30 (↑), miR-186* (↓), miR-206 (↑), miR-21 (↓), miR-874 (↑), miR-let7c (↑), miR-101 (↑), CIRC-PRKCA (↓) miR-384 (↑)	MTA1, CCND1/Wnt1/MYC, MMP2, MMP9, ATP1B1, PI3K/Akt signaling pathway, c-MYC, PTEN, ITGB1	Reduced their invasion, inhibited their migration	[110]

Note: ↑: upregulation; ↓: downregulation.

**Table 4:** Clinical application of polyphenols.

Polyphenol	Compound	NCT Number	Title	Status	Phase	Population
Flavonoids	EGCG	NCT01317953	Oral Green Tea Extract for Small Cell Lung Cancer	Available	-	-
	EGCG	NCT02577393	Study of Epigallocatechin-3-gallate (EGCG) for Esophagus Protection in Patients with Lung Cancer Receiving Radial Radiotherapy	Enrolling by invitation	2	83
	EGCG	NCT00573885	Green Tea Extract in Preventing Cancer in Former and Current Heavy Smokers with Abnormal Sputum	Completed	2	53
	EGCG	NCT00611650	Green Tea Extract in Treating Current or Former Smokers with Bronchial Dysplasia	Terminated	2	23
	EGCG	NCT04871412	The Thoracic Peri-Operative Integrative Surgical Care Evaluation Trial Stage II	Not yet recruiting	3	40
	Isoquercetin	NCT02195232	Cancer Associated Thrombosis and Isoquercetin (CATIQ)	Completed	2/3	64
	Isoflflavones	NCT01958372	Radiation Therapy, Chem motherapy, and Soy Isoflavones in Treating Patients with Stage IIIA-IIIB Non-Small Cell Lung Cancer	Completed	1	11
	Genistein	NCT01628471	MTD Determination, Safety and Efficacy of the Decitabine-Genistein Drug Combination in Advanced Solid Tumors and Non-Small Cell Lung Cancer	Completed	1/2	20
	Chlorogenic acid	NCT03751592	Phase Ib/IIa Studies of Chlorogenic Acid for Injection for Safety and Efficacy of Advanced Lung Cancer	Unknown status	1/2	144
Phenolic acids	Black Raspberry	NCT04267874	Black Raspberry Nectar for the Prevention of Lung Cancer, BE WELL Study	Active, not recruiting	1	96
Curcuminoids	Curcumin C3 complex	NCT03598309	Phase II Trial to Modulate Intermediate Endpoint Biomarkers in Former and Current Smokers	Recruiting	2	75
	Blueberry powder	NCT01426620	Standard Chemotherapy with Blueberry Powder in Non-Small Cell Lung Cancer	Terminated	2	4

## Data Availability

Not applicable.
